# A Novel Friction Stir Deposition Technique to Refill Keyhole of Friction Stir Spot Welded AA6082-T6 Dissimilar Joints of Different Sheet Thicknesses

**DOI:** 10.3390/ma15196799

**Published:** 2022-09-30

**Authors:** Mohamed M. Z. Ahmed, Mohamed M. El-Sayed Seleman, Essam Ahmed, Hagar A. Reyad, Naser A. Alsaleh, Ibrahim Albaijan

**Affiliations:** 1 Mechanical Engineering Department, College of Engineering at Al Kharj, Prince Sattam Bin Abdulaziz University, Al Kharj 11942, Saudi Arabia; 2 Department of Metallurgical and Materials Engineering, Faculty of Petroleum and Mining Engineering, Suez University, Suez 43512, Egypt; 3 Department of Mechanical Engineering, College of Engineering, Imam Mohammad Ibn Saud Islamic University, Riyadh 11432, Saudi Arabia

**Keywords:** aluminum alloys, AA6082-T6, AA2011-T6, friction stir spot welding, friction stir deposition, keyhole refilling, mechanical properties

## Abstract

Joining dissimilar sheet thicknesses of AA6082-T6 alloys by friction stir spot welding (FSSW) provides many advantages in automotive and aerospace applications. The formed keyhole at the end of the FSSW process is one of the typical features after the welding process, which owns the same size as the rotating pin that remains at the joint center. This keyhole destroys the joint continuity and can stimulate serious stress concentration when the FSSW joint bears an external force. To solve this issue, a novel refilling technique was developed for the FSSW keyholes using a friction stir deposition (FSD) technique. The FSSW joints of AA6082-T6 sheets were welded at various rotation speeds from 400 to 1000 rpm and a constant dwell time of 3 s, where a 2 mm sheet thickness was an upper sheet, and a 1 mm sheet thickness was a lower sheet. All the keyhole refilling processes were achieved using a specially designed AA2011-T6 consumable rod to be used for friction stir deposition of continuous layers at a constant deposition parameter of 400 rpm consumable rod rotation speed and a 1 mm/min feed rate. The heat input energy for both the FSSW and refilled FSSW lap joints was calculated. In addition, the FSSW and the FSD temperatures were measured. Macrostructure, microstructure, and mechanical properties in terms of hardness and tensile shear maximum load were evaluated for both the friction stir spot welded (FSSWed) and the refilled FSSW lap joints. The obtained results showed that the keyhole could be successfully refilled with defect-free continuous multilayers after the refill friction stir spot welding (RFSSW) process. All the RFSSW lap joints showed higher tensile shear loads than that given by the FSSW (before refill) lap joints. The RFSSW joint (welded at 600 rpm/3 s and refilled at 400 rpm/1 mm/min) showed a higher tensile shear load of 5400 N ± 100 compared with that recorded by the unrefilled joint (4300 N ± 80). The fracture location and fracture surface of the FSSW and RFSSW were examined and discussed.

## 1. Introduction

Lightweight components are essential in the automotive and aerospace industries for the reduction of CO_2_ emissions. The usage of aluminum alloys can reduce the vehicle structure’s total weight [1,2]. Different spot joining techniques have been used in the automotive industry, such as riveting and resistance spot welding. Recently, to avoid the drawbacks of these spot welding techniques, alternative joining techniques in terms of friction stir welding (FSW) and its derivative friction stir spot welding (FSSW) processes are used for joining similar [3,4] and dissimilar structural materials [5,6,7,8,9] in engineering applications. The FSSW is a solid-state joining technique. It is achieved by applying a controlled thermomechanical process to produce spot lap joints based on the generated frictional heating and plastic deformation in the stir zone [10,11,12]. The quality of the friction stir spot welded (FSSWed) joints is controlled by welding process parameters, such as tool rotation speed [13,14], dwell time [1,15], plunge rate [16], axial downward force [17], and tool design [18,19]. The tool rotation speed plays a vital role in the frictional heat generation, whereas the downward force, plunging rate, dwell time, and tool design are credited to the material flow at the spot joint interface [20]. The most serious defect for the FSSWed joints is keyhole formation [21,22]. Nowadays, modified FSSW processes are developed to eliminate the keyhole defect, such as pinless friction stir spot welding [1,15], flat friction stir spot welding [23,24], and refill friction stir spot welding (RFSSW) [22,25,26]. For the pinless friction stir spot welding, the rotating tool has no pin, and its shoulder surface is recommended to be a scroll groove [1,15]. This process is only applicable to a weld aluminum sheet thickness of less than 2 mm [1]. The flat friction stir spot welding uses two different tools (a tool with a pin and a pinless tool) for producing the FSSW joint and refilling the formed keyhole [23,24]. In the first step, a specially designed back plate with a predrilled dent is utilized to yield a protuberance on the bottom side of the joint. In the second step, the protuberance and keyhole are removed by applying a pinless rotating tool and a smooth flat back plate [23]. This technique is complex and requires many precautions in design and implementation; in addition, it depends on the deformability of the welded materials. The RFSSW process contains four stages, which are friction heating, plunging, refilling, and joint forming; in addition, its tool rotation is complicated and mainly consists of three independent parts [22,25,26]. The first part is a clamping ring, which is used to retain the sheets and prevent the viscous plastic material from escaping. The other two parts are a sleeve and a pin; both are moved independently in opposite vertical directions to each other to realize the stirring and the refilling of the viscous plastic material. At the end of the process, the displaced material is initially pushed to the surface level, without the keyhole on the sheet surface. Furthermore, the process temperature may reach the melting point, and the formation of liquation cracks is difficult to avoid [27]. These complications have limited the use of this technique in many engineering applications.

Recently, friction stir deposition (FSD) as an additive manufacturing technology is recommended by many authors to build multilayers of different materials on various substrates [28,29,30]. This technology is a suitable technique to produce metallic parts for the automobile and aircraft industries. It is also a thermomechanical process based on the FSW principles, by adding a mechanism of material feeding to deposit alloys [29,30,31] and composites [28]. Perry et al. [32] concluded that the FSD path of AA2024 Al alloy at a 300 rpm tool rotational speed, 2 mm/s travel speed, and 0.85 mm/s feed rate reveals an almost fully recrystallized microstructure. Dilip et al. [33] investigated the microstructure of an AA2014-T6 friction stir deposited at an 800 rpm tool rotation speed, 1 mm/min feed rate, and 35 MPa axial pressure. The results showed that fine grains and refined second-phase precipitates are the microstructure features. Priedeman et al. [34] evaluated the microstructure features and hardness of FSD 110 Cu processed at a travel speed of 2.12 mm/s and rotational speed of 275 rpm. The results showed that the microstructure features are recrystallized fine grains throughout the deposited layers, with different degrees of grain refining; in addition, the hardness measurement showed that the deposited layers are softer than that of the base material. 

Based on the literature review, although there is a great interest to publish many works in FSD, there is no attempt to use FSD in refilling the FSSW keyhole. Thus, the current study is considered the first try in the world to apply the FSD process for filling and repairing keyholes of the FSSW joints of AA6082-T6 with different sheet thicknesses produced at a constant dwell time of 3 s and different rotation speeds of 400, 600, 800, and 1000 rpm. A specially designed consumable rod of AA2011-T6 was suggested. The heat input energy at applied deposition process parameters of 400 rpm consumable rod rotation speed and 1 mm/min feed rate was studied, and a new equation to calculate the heat input energy was suggested. The current work aims also to conduct a comparative study of the FSSW and the RFSSW lap joints in terms of macrostructure, microstructure, hardness, maximum tensile shear, and fracture behaviors.

## 2. Materials and Methodology

### 2.1. Starting Materials

The starting materials to achieve dissimilar thickness friction stir spot welds were two thin sheets of AA6082-T6 with dimensions of 1 × 1000 × 1000 mm and 2 × 1000 × 1000 mm. Meanwhile, the keyhole filling material was AA2011-T6 rods with initial dimensions of 20 mm diameter and 100 mm length. Both the AA6082-T6 sheets and the AA2011-T6 rods were supplied by the Future Fond Company, Milano, Italy. The chemical compositions of the used AA6082-T6 sheets and the AA2011-T6 rods were performed using Foundry-Master Pro, Oxford Instruments, Abingdon, UK, and are listed in Table 1.

### 2.2. Friction Stir Spot Welding and Friction Stir Deposition Processes

**Figure 1:**
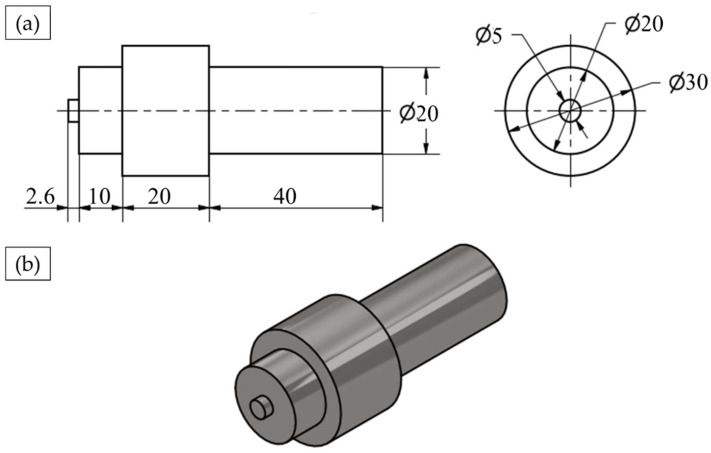
(**a**) Schematic of welding tool (all dimensions in mm) and (**b**) isometric view of designed FSSW tool.

The FSSW joints and the FSD refilled samples were carried out using the friction stir welding/processing machine (EG-FSW-M1) [35]. For the spot welding process, the two AA6082-T6 dissimilar sheets were cut to specimens with dimensions of 30 mm width and 100 mm length. The cut specimens were FSSWed in lap joints at a constant dwell time of 3 s and various rotational speeds of 400, 600, 800, and 1000 rpm, where the two different sheet thicknesses overlapped with a 30 mm length. The other FSSW parameters in terms of plunge rate and plunge depth were kept constant at 0.1 mm/s and 2.6 mm, respectively. The used FSSW tool was made of steel (AISI H13) with dimensions of 20 mm shoulder diameter, 2.6 mm pin length, and 5 mm pin diameter. The tool shoulder was flat, and the pin was cylindrical. The tool features and dimensions are given in Figure 1. 

**Figure 2:**
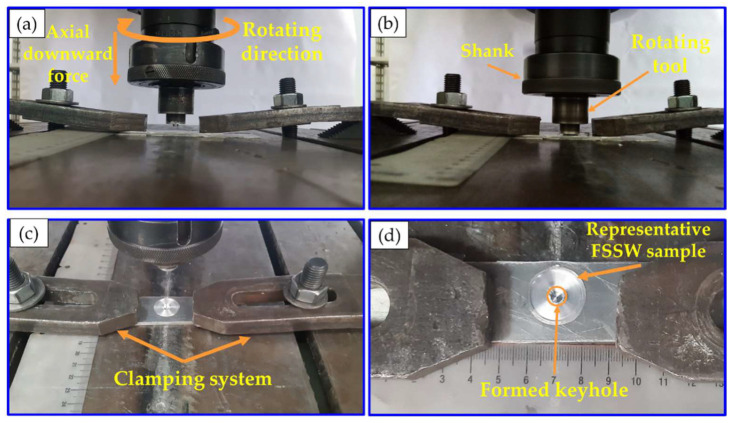
(**a**–**c**) summarizes the stages of the FSSW process of dissimilar sheet thickness AA6082-T welds. (**d**) shows the produced joint top view with the formed keyhole [36].

**Figure 3:**
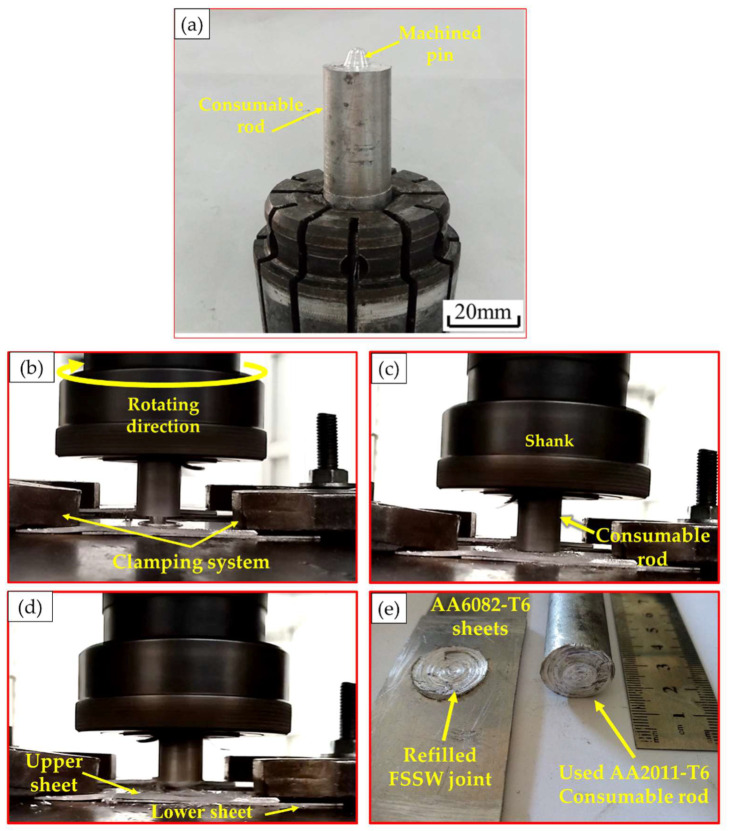
(**a**) Image shows the fixing of the machined AA 2011-T6 consumable rod for FSD, (**b**–**d**) summarizes the stages of the FSD process, and (**e**) shows the top view of the refilled keyhole FSSW dissimilar lap joint.

Figure 2 illustrates the FSSW process stages, which involve three main steps: (1) fixing the lap joint specimens with a clamping system (Figure 2a), (2) the plunging and stirring stage with the rotating tool (Figure 2b), and (3) the retracting stage (Figure 2c). Finally, Figure 2d shows the representative spot-welded joint’s top view with the formed keyhole. The temperature during the FSSW process at the applied different rotational speeds of 400 to 1000 rpm was recorded using a modern digital multimeter (type-UT61B, Zhejiang, China) with a thermocouple type “K”.

In order to explore the applicability of FSD for refilling the left keyhole after FSSW, an AA2011-T6 consumable rod was machined (according to the keyhole volumetric dimension) to a profile of a flat shoulder of 20 mm diameter and a tapered pin of 5 mm diameter and 3 mm pin length (as shown in Figure 3a) to be tried as a filling material in continuous layers via the FSD process. Figure 3 illustrates the FSD process stages, which involve three steps: fixing the AA 2011-T6 consumable rod in the spindle shank (Figure 3a) and rotating it at a constant rotation of speed 400 rpm while moving downward to reach the FSSW keyhole of the AA6082-T6 joint (Figure 3b). Finally, under a continuous feeding speed of 1 mm/min, the rod plastically deformed due to the high friction and the generated heat between the rod and the keyhole substrate, causing the material transfer from the consumable rod to the keyhole to build a material upward yielding refilling. Figure 3e shows the representative top view of the refilled FSSW joint.

**Figure 4:**
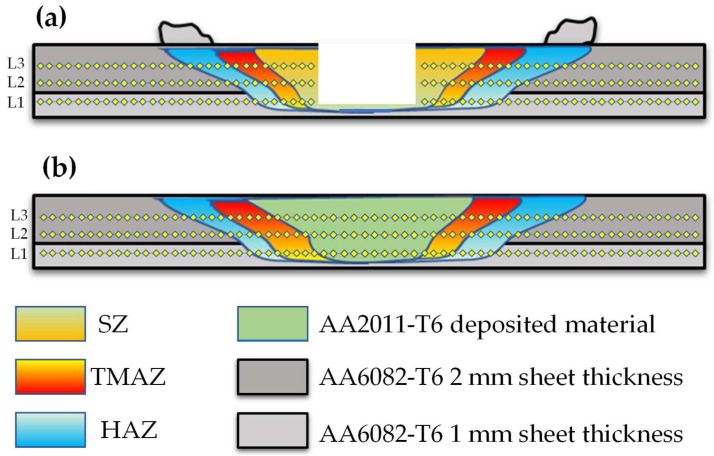
Schematics for the positions of the hardness test lines: (**a**) FSSWed AA6082-T6 dissimilar sheet thickness joint and (**b**) RFSSWed joint (all dimensions in mm).

**Figure 5:**
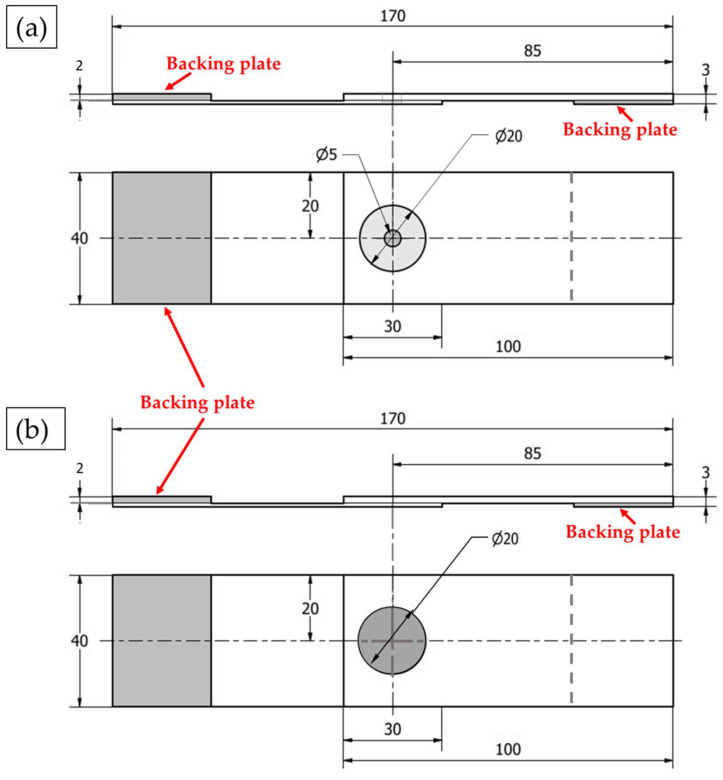
Schematic of tensile-shear test specimens: (**a**) the FSSWed AA6082-T6 dissimilar sheet thickness joint and (**b**) the RFSSWed joint (all dimensions in mm).

The produced FSSWed and refilled friction stir spot welded (RFSSWed) joints were sectioned for macrostructure investigation, microstructure evaluation, and hardness test. The cross-sectional specimens were ground with SiC papers up to 2400 grit, then polished using a polishing vel-cloth in the presence of alumina paste suspension up to a surface finish of 0.05 µm. A chemical etcher consisting of 2.5 mL nitric acid (HNO_3_), 1.5 mL hydrochloric acid (HCl), 95 mL distilled water, and 1 mL hydrofluoric (HF) was applied to all the polished specimens before microstructure investigation. The microstructure evaluation was performed using an Olympus optical microscope (OM) (BX41M-LED, Olympus, Tokyo, Japan). The hardness test was carried out along three lines across the transverse sections of the FSSWed and the refill friction stir spot welded (RFSSWed) joints for obtaining hardness profiles (Figure 4). The hardness measurements were carried out using an applied load of 5 N with a dwell time of 15 s using a Vickers hardness tester (model HWDV-75, TTS Unlimited, Osaka, Japan). The distance between every two indentations was set to 0.5 mm along the cross section of the produced FSSWed and RFSSWed joints. The tensile-shear test was performed at room temperature using a 30 ton universal tensile testing machine (Type-WDW-300D, Guangdong, China) at a constant loading rate of 0.1 mm/min. During the tensile-shear test, two backing sheets were used to ensure the application of the axial loading (Figure 5). After tensile shear testing, the fractured surfaces at the interface between the upper and lower sheets were examined using a scanning electron microscope (SEM-Quanta FEG 250-FEI Company, Hillsboro, OR, USA). For comparison purposes, the as-received materials were examined and tested using the previous described methods.

## 3. Results and Discussion

### 3.1. FSSWed and RFSSWed Joint Surfaces’ Appearance

**Figure 6:**
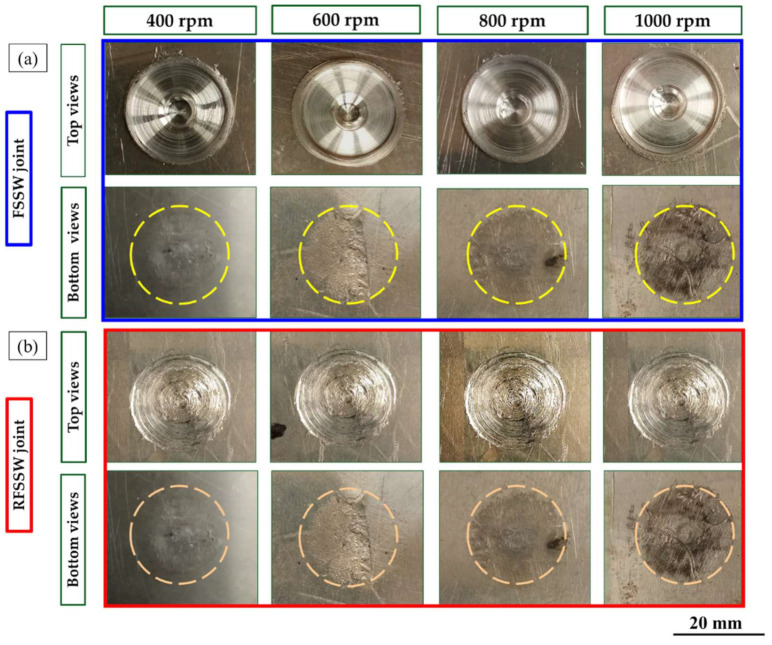
The visual appearance of the top and bottom surface views of (**a**) the AA6082-T6 FSSW joints FSSWed at a constant dwell time of 3 s and different rotation speeds of 400, 600, 800, and 1000 rpm and (**b**) the RFSSW joints after refilling the keyholes.

The top and bottom surfaces of the produced FSSWed AA6082-T6 dissimilar sheet thickness and their RFSSWed joints with AA2011-T6 deposited layers at the applied processing parameters (FSSW and refill processing parameters) are shown in Figure 6. The top and bottom surfaces of the FSSW lap joints welded at different rotation speeds of 400, 600, 800, and 1000 rpm and a constant dwell time of 3 s are represented in Figure 6a. Meanwhile, the top and the bottom surfaces of the RFSSW joints processed at a constant consumable rod rotation speed of 400 rpm and a constant feed rate of 1 mm/min are shown in Figure 6b. It can be remarked that the applied welding parameters for joining the dissimilar thicknesses of the AA6082-T6 thin sheet are appropriate to produce successful spot joints. The circular indentations are shown at the top surface views of the FSSW lap joints, indicating the shoulder projection for all the applied welding parameters. The sizes of the extruded flash materials are also the same (Figure 6a). Furthermore, the bottom surface views of the FSSW lap joints show the affected areas (dark areas) by thermal exposure due to the FSSW process (as shown in Figure 6a). These thermal affected areas become darker by increasing the rotation speeds from 400 to 1000 rpm at a constant dwell time of 3 s due to the increase in heat input generation with increasing rotation speed [6,36], as seen in Figure 6a.

Figure 6b shows the top surface appearance of RFSSWed joints with FSD process parameters of 400 rpm consumable tool rotation speed and 1 mm/min constant feeding rate. Visually, it can be remarked that defect-free refilling using AA2011 material top surfaces was attained for all the processed joints. It can be concluded that the suggested FSD parameters succeeded to refill all the keyholes and the shoulder projections of the FSSW lap joints. In addition, there was no remarkable effect of the exposure thermal cycle during FSD on the bottom surface appearance of the RFSSW lap joints (Figure 6b) compared to that given by the unrefilled FSSW joints (Figure 6a).

### 3.2. Heat Input Energy Calculations and Peak Temperature Measurements

The total heat input energy is the summation of the generated heat input energy at the contact shoulder area and the pin surrounding contact area. The generated heat input energy during the FSSW process depends on the tool design, tool material, friction axial downward force, friction coefficient at the interface between the rotating pin and the surrounded stirring material, applied dwell time, and rotation speed [9,37]. The heat input is generated by converting the mechanical energy from the friction of the rotating tool through the joint thickness during the FSSW thermomechanical process. The heat input energy for the FSSW process (*Q*) can be calculated based on the following equations [9,38]:

(1) $$Q=1.083\times \mu \times \frac{P}{K_{A}}\times \omega \times r\times t$$

where μ is the friction coefficient between the steel tool and the aluminum alloy sheet, which equals 0.4 [4]; P is the applied downward force (in N); $K_{A}$ is the ratio of the shoulder contact area to cross-sectional area of the rotating tool; ω is rad/s and equals $\frac{\pi }{30}n$, where n is the applied rotational speed (in rpm); *r* is the pin radius (in m); and *t* is the dwell time (in s) during the FSSW process:

(2) $$K_{A}=\frac{\left(\mathrm{shoulder}\;\mathrm{radius}\;\mathrm{of}\;\mathrm{the}\;\mathrm{rotating}\;\mathrm{tool}\right){\;}^{2}-{\left(\mathrm{pin}\;\mathrm{radius}\right)}^{2}}{{\left(\mathrm{shoulder}\;\mathrm{radius}\;\mathrm{of}\;\mathrm{the}\;\mathrm{rotating}\;\mathrm{tool}\right)}^{2}}$$

where the shoulder radius of the rotating tool and pin radius is equal to 10 and 2.5 mm, respectively. Then $K_{A}=$ 0.9375. From Equations (1) and (2), the calculated heat input energy is as follows:

(3) $$Q=1.1859\times 10^{\left(-3\right)}\times P\times n\times t\;(J)$$

**Figure 7:**
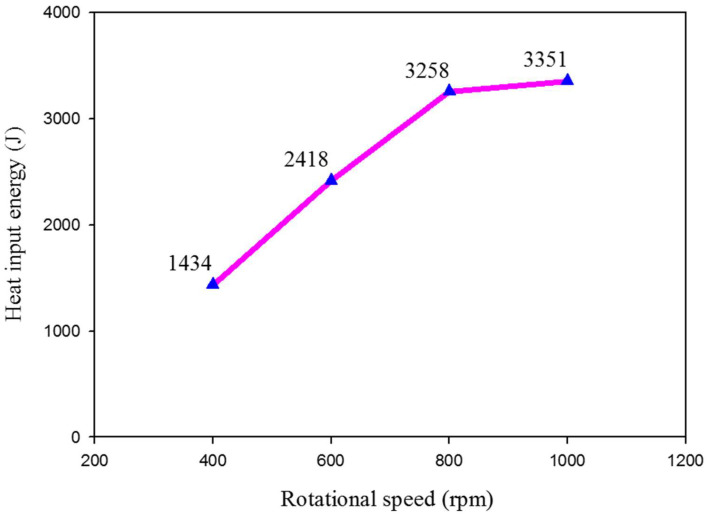
The calculated heat input energy against the applied rotational speeds of the FSSWed AA6082 joints.

**Figure 8:**
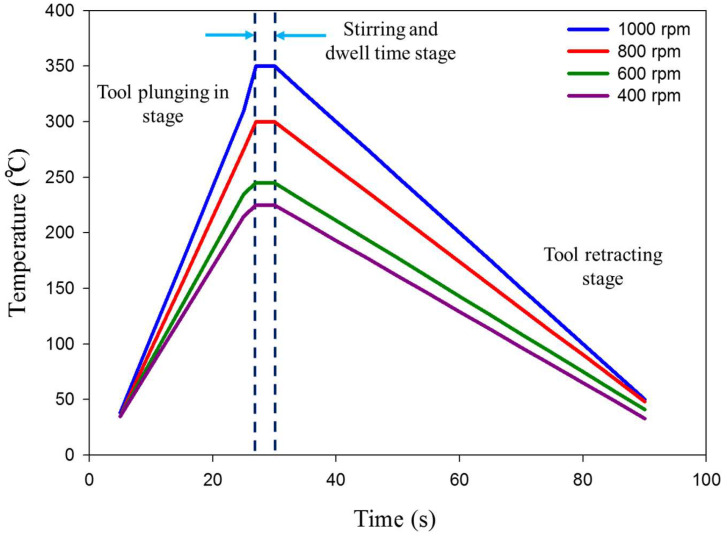
Temperature—time curves (thermal cycles) during FSSW of the dissimilar sheet thickness AA6082-T6 joints welded at different rotational speeds of 400, 600, 800, and 1000 rpm while keeping the dwell time constant (3 s).

The calculated *Q* of the FSSW joints is plotted against the applied rotation speed, as given in Figure 7. It can be noted that the increase of the rotation speeds from 400 to 1000 rpm leads to an increase in the generated heat input energy from 1434 to 3351 J during the FSSW process [39]. The FSSW thermal cycle exposure and the measured temperatures of the produced FSSW lap joints are shown in Figure 8.

The thermal cycles of the FSSW AA6082-T6 lap joint in terms of the measured working temperature against the consuming time to yield the spot joint at the applied rotation speeds of 400 to 1000 rpm are given in Figure 8. It can be observed that the experienced thermal cycle for all the processed joints gives the same trends at the welding parameters, with difference in the peak temperatures at each tool rotation speed. Each thermal cycle involves three stages, including rising temperature gradually by inserting the rotating tool pin at a constant feeding rate of 0.1 mm/s through the clamping of two different sheet thicknesses of AA6082-T6 (first stage). The second stage represents the dwell time required to perform the spot weld at near temperature stability. The last stage represents rotating tool retracting at the end of the spot-welding process. Table 2 illustrates the measured peak temperature in the stir zone (SZ) during the FSSW process. It can be noted that the peak temperature increases from to 225 ± 2 to 350 ± 2 with the increasing tool rotation speed from 400 to 1000 rpm, respectively, as a result of frictional heat during the stirring process [39]. 

Based on the friction stir processing [40,41,42] and deposition [43,44] concepts, the total heat input (*Q_t_*) to refill the FSSW keyhole via FSD is the summation of the frictional heat generated by the consumable tool shoulder between the rotating consumable pin and the surrounded material in the vicinity of the keyhole and at the consumable pin tip. In general, the heat generation is given as follows:

(4) $$dQ=\omega \times dM=\omega \times r\times dF=\omega \times r\times \tau_{contact}\;dA$$

(5) $$\tau_{contact}=\tau_{friction}=\mu \times P=\mu \times \frac{F}{A}$$

P is the contact pressure at friction interfaces, and it is equal to the axial downward force divided by the projected areas.

The heat input generation under the consumable rod shoulder (*Q*_1_) is calculated by integration Equation (4).

(6) $$Q_{1}=\int_{0}^{2\pi }\int_{R_{P}}^{R_{s}}\omega \times r^{2}\times \tau_{friction}\times d\theta \times dr=\frac{2}{3}\times \pi \times \omega \times \mu \times \frac{F}{A}\times \left(r_{s}^{3}-r_{p}^{3}\right).$$

The heat input generation between the rotating consumable pin and the surrounded material in the vicinity of the keyhole (*Q*_2_) is calculated by integration Equation (4).

(7) $$Q_{2}=\int_{0}^{2\pi }\int_{0}^{H_{p}}\omega \times r_{p}^{2}\times \tau_{friction}\times d\theta \times dz=\frac{2}{3}\times \pi \times \omega \times \mu \times \frac{F}{A}\times r_{p}^{2}\times H_{p}$$

The heat input generation at the consumable pin tip (*Q*_3_) is calculated by integration Equation (4).

(8) $$Q_{3}=\int_{0}^{2\pi }\int_{0}^{R_{p}}\omega \times r_{p}^{2}\times \tau_{friction}\times d\theta \times dr=\frac{2}{3}\times \pi \times \omega \times \mu \times \frac{F}{A}\times r_{p}^{3}$$

The total heat input energy (*Q_t_*) is calculated by summations of Equations (5)–(8):

$$Q_{t}=Q_{1}+Q_{2}+Q_{3}$$

(9) $$Q_{t}=\frac{2}{3}\times \pi \times \omega \times \mu \times \frac{F}{A}\times \left(r_{s}^{3}+r_{p}^{2}\;H_{p}\right)$$

(10) $$Q_{t}=\frac{2}{3}\times \mu \times \omega \times F\times \left(r_{s}+3\;\frac{H_{p}\times r_{p}^{2}}{r_{s}^{2}}\;\right)$$

where $Q_{t}$ is in watt, μ is the friction coefficient between AA6082-T6 keyhole material and AA2011-T6 consumable rod material and equals 1.4 [45], ω is in rad/s and equals $\frac{\;\pi \;n}{30}$, n is the rotational speed (in rpm), F is the axial downward force (in N), $r_{s}$ is the consumable rod shoulder radius (in m), $r_{p}$ is the consumable rod pin radius (in m), $H_{p}$ is the consumable rod pin height (in m), and t is the deposition time (in s).

$$Q_{t}=\frac{2\pi }{90}\times \mu \times n\times F\times \left(r_{s}+3\;\frac{H_{p}\times r_{p}^{2}}{r_{s}^{2}}\;\right)$$

$$Q_{t}=2250\;W$$

The refilling time of FSD = refill height ×60=180 s.

The total heat input energy (QE) = $Q_{t}\times t$ (in J).

**Figure 9:**
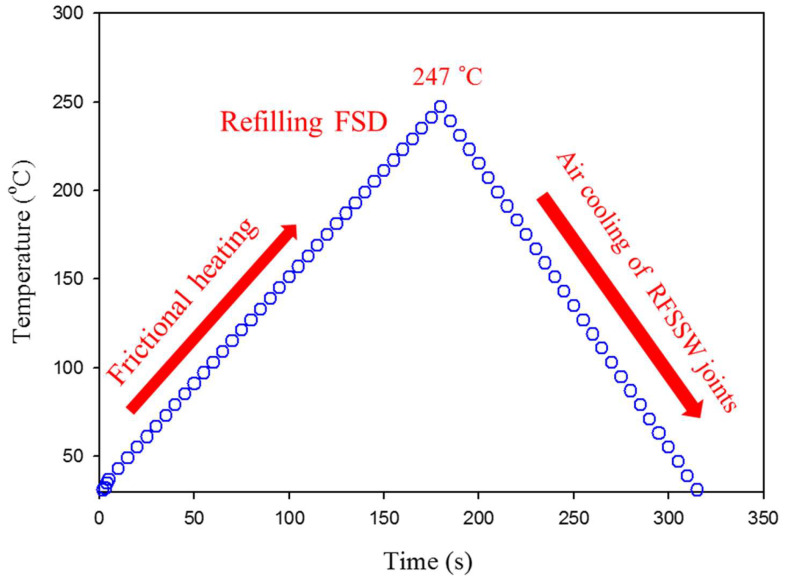
The temperature measurements against time during the FSD process.

The knowledge of the thermal cycle in terms of the heat generation and the temperature history during the FSD process is very important in understanding the thermomechanical interaction occurring during the deposition process in the keyhole of the FSSW joint. The heat generates at the contact area between the consumable rod and the substrate (keyhole bottom) due to the friction during the stirring process. The heat to develop a bond between the keyhole bottom and the deposited material involves transferred heat to the FSD consumable tool, transferred heat to the keyhole bottom, and remaining heat for depositing a material [43]. When the generated peak temperature during the FSD process is below the melting point of the deposition material, the deformation is a solid-state process [43,46]. The thermal cycle to achieve refilling of the FSSW joints of AA6082-T6 is represented as temperature versus time, as given in Figure 9. It can be noted that the deposition peak temperature is 247 °C, which is below the melting point of the AA2011-T6 deposited material. This solid-state process promotes good bonding between the AA2011-T6 deposited material and the keyhole bottom of FSSW dissimilar thickness joints. A similar finding in terms of good bonding between the deposited materials and the substrates is reported by other researchers to build sound continuous layers of as-cast hypoeutectic A356 Al alloy on the AA2024 substrate [30] and also sound continuous layers of AA2011 in two temper conditions on the AA5083 substrate [29] without any defects at the interface. 

### 3.3. Macrographs of the FSSW and RFSSW Lap Joints

**Figure 10:**
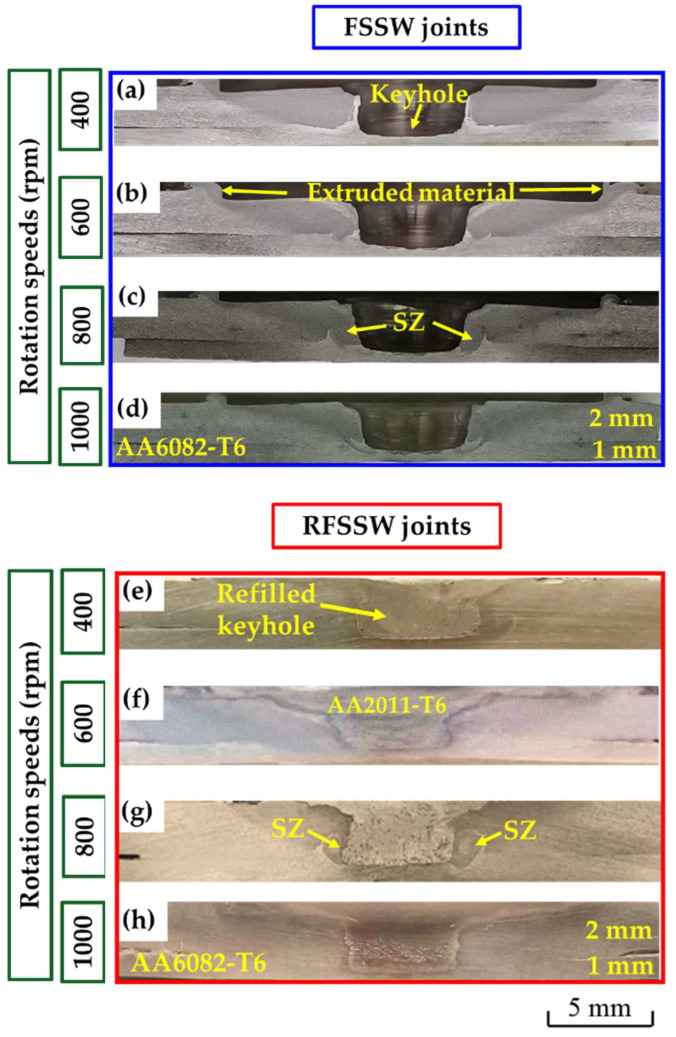
Transverse cross-section macrographs of (**a**–**d**) the FSSW lap joints of AA6082-T6 welded at a constant dwell time of 3 s and different rotational speeds of 400 to 1000 rpm [36] and (**e**–**h**) RFSSW deposited at a 400 rpm rotation speed and a 1 mm/min deposition rate.

Figure 10 illustrates the transverse macrographs of the FSSW AA6082-T6 dissimilar sheet thickness joints and their RFSSW counterparts. It can be seen that the applied FSSW parameters resulted in defect-free welds at the interface of the overlapped 1 mm and 2 mm AA6082-T6 sheets (Figure 10a–d). In addition, the excessive extruded flash material was eliminated as a result of the FSSW optimization welding parameters based on some experiments and published works [3,6]. The resulting spot welded joint has a characteristic hole in the middle of the joint; this hole is left by the pin after removal. This keyhole (material loss) is the main defect in the FSSW process, and it is a good representative of the pin dimensions and geometry (Figure 10a–d). Figure 10e–h represents the cross-section macrographs of the RFSSW joints. The keyhole refilling process was achieved at the deposition conditions of 400 rpm consumable rod rotation speed and 1 mm/min deposition rate. It can be remarked that the keyhole can be successfully refilled by the FSD process using the deposition material of AA2011-T6. Furthermore, the deposited materials bonded well with the keyhole inner surface without porosity and microcracks for all the refilled joints. 

### 3.4. Microstructure Investigation of the Base Materials

**Figure 11:**
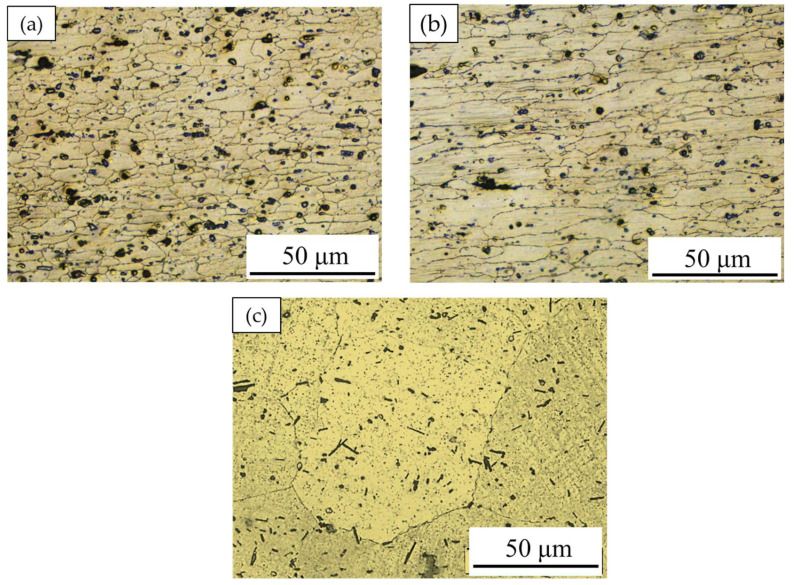
OM microstructure images of the starting BMs: (**a**) 1 mm, (**b**) 2 mm AA6082-T6, and (**c**) AA2011-T6 [29].

The microstructures of the base materials (BMs) for the two AA6082-T6 sheet thicknesses at 1 and 2 mm and the AA2011-T6 rod Al alloys are shown in Figure 11. It can be seen that the microstructures of the AA6082-T6 BMs show coarse and elongated grain structures, indicating the rolling directions. In addition, they contain comparatively coarse (Fe, Mn)_3_SiAl_l2_ intermetallic and low-density fine dispersed Mg_2_Si precipitates. These microstructure features are consistent with that reported in other works [2,36,47]. It can be observed also that the AA 6082-T6 sheets of 1 (Figure 11a) and 2 mm (Figure 11b) thickness have average grain sizes of 11 ± 2 and 17 ± 1.5 μm, respectively. The variation in the grain size values of the two sheets can be ascribed to imposing different rolling conditions to produce different sheet thicknesses. This reduction in thickness as a result of high accumulated plastic deformation promotes high internal strain, which increases the strength [48]. As reported in [28,29], the microstructure of the AA2011-T6 rod alloy shows coarse grains and the intermetallics of Al_7_Cu_2_Fe (rodlike shape) and Al_2_Cu (almost spherical or irregular shapes), as shown in Figure 11c.

### 3.5. Hardness Results Evaluation

**Figure 12:**
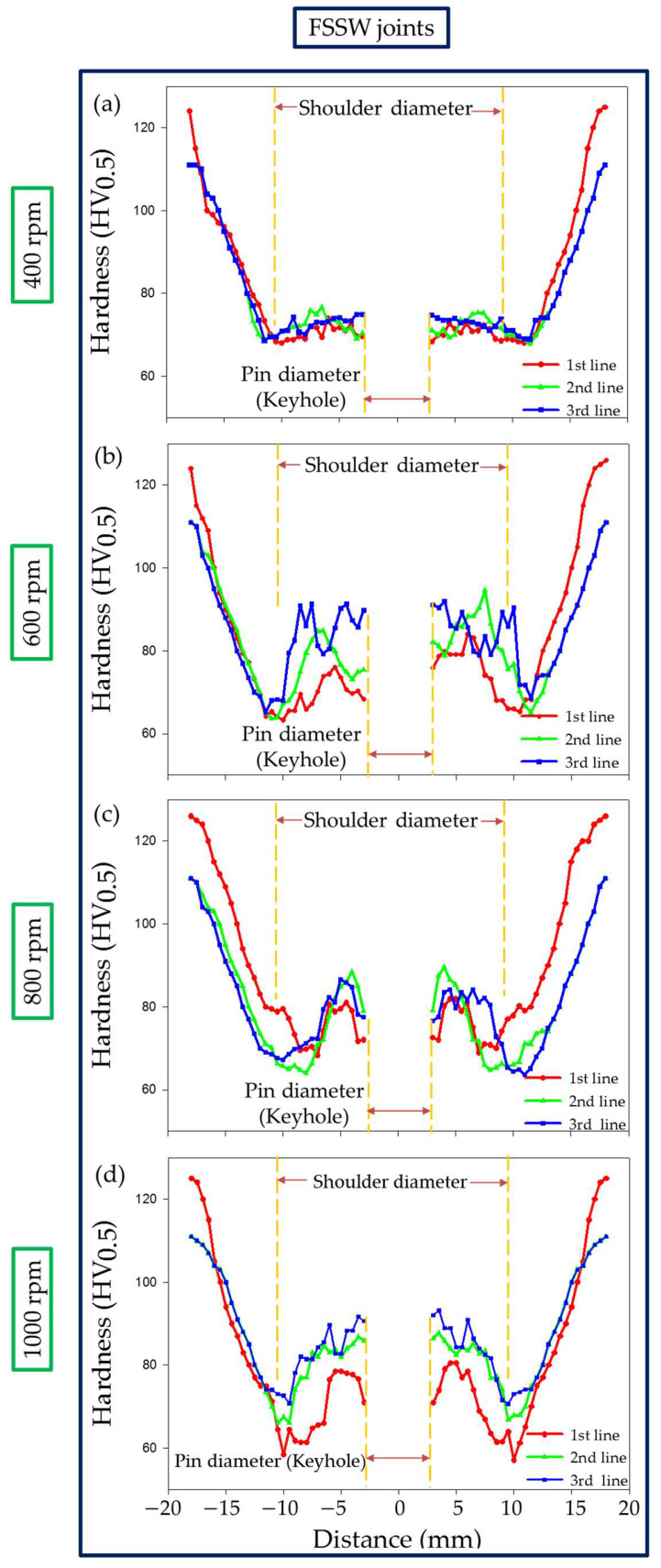
Hardness profile of the dissimilar sheet thickness AA6082-T6 FSSW joints produced at (**a**) 400 rpm, (**b**) 600 rpm, (**c**) 800 rpm, and (**d**) 1000 rpm rotation speeds and a constant dwell time of 3 s.

**Figure 13:**
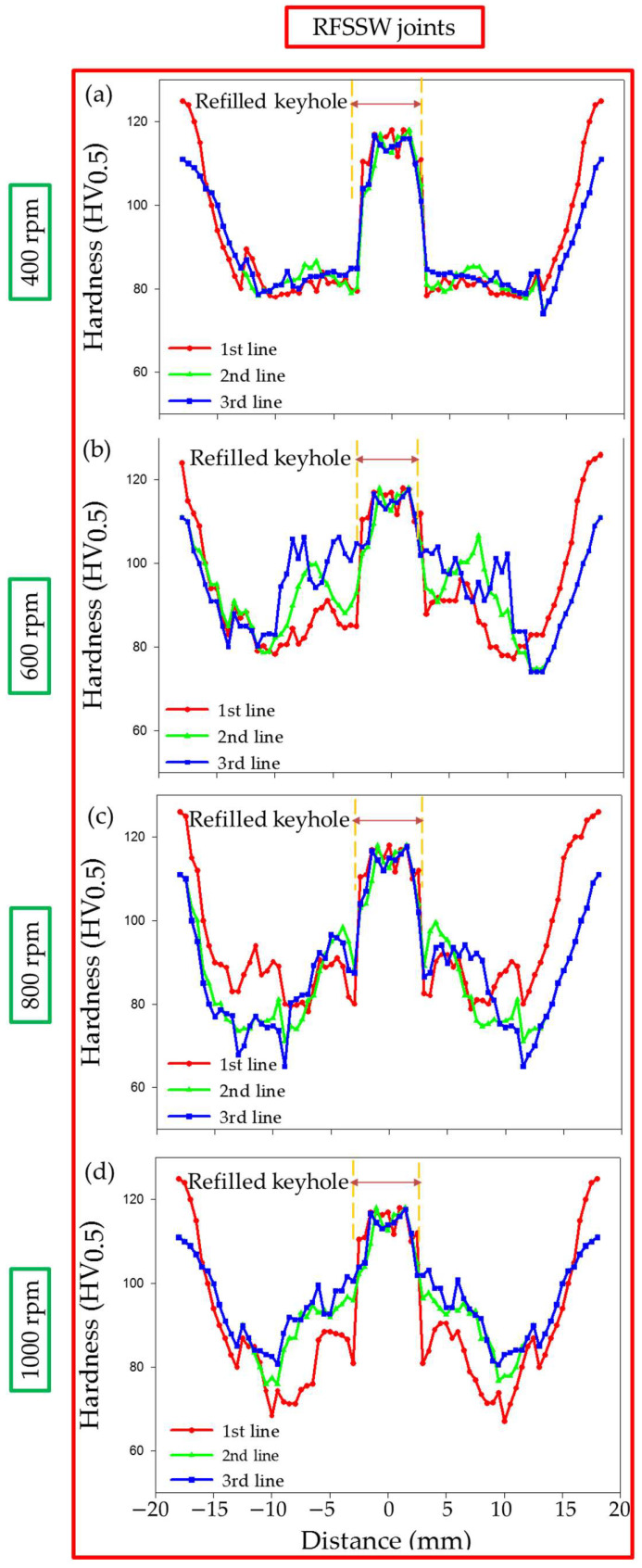
Hardness profile of the AA6082-T6 RFSSW joints spot welded at (**a**) 400 rpm, (**b**) 600 rpm, (**c**) 800 rpm, and (**d**) 1000 rpm rotation speeds with applying a constant dwell time of 3 s and refilled with AA2011-T6 via the FSD process at a 400 rpm rotation speed and a 1 mm/min feed rate.

Hardness measurements were carried out on the transverse cross sections of both the AA6082-T6 FSSW joints of the dissimilar sheet thickness and RFSSW joints and plotted in a hardness profile. Figure 12 shows the hardness profile for the FSSW joints produced at a constant dwell time of 3 s and different rotation speeds of 400, 600, 800, and 1000 rpm. Meanwhile, Figure 13 represents the hardness profile of the RFSSW joints, refilled with AA2011-T6 via the FSD process at a 400 rpm rotation speed and a 1 mm/min feed rate.

**Figure 14:**
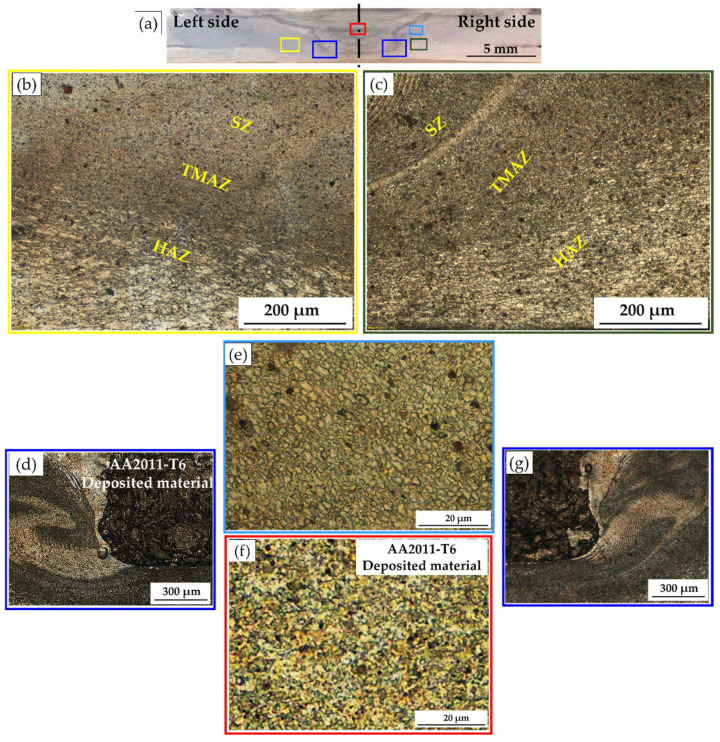
(**a**) Macrostructure image of the AA6082-T6 RFSSWed joint welded at 600 rpm and 3 s with keyhole refilling by AA2011-T6 via the FSD conditions of 400 rpm and 1 mm/min. And microstructure images of (**b**) the spot weld zones on the left side, (**c**) the spot weld zones on the right side, (**d**) weld zones/deposited material interface on the left side, (**e**) the SZ of the welded joint, (**f**) SZ of the deposited material and (**g**) weld zones/deposited material interface in the right side.

Compared with the different sheet thickness AA6082-T6 BMs, the hardness of the weld zones, the SZ, thermomechanically affected zone (TMAZ), and the heat-affected zone (HAZ) of the FSSWed joints at the used rotation speeds of 400, 600, 800, and 1000 rpm significantly decreased, as shown in Figure 12a–d, respectively, due to annealing of the AA6082-T6 BMs during the FSSW process. It was reported that the hardness values across the weld zones of the joints of heat-treatable Al alloys are affected by thermal exposure during FSW [36,49]. Among the weld zones for each FSSWed joint, the minimum hardness values were observed in the HAZ due to the grain structure and overaging effect [36]. In contrast, the hardness improvement in the SZ is mainly due to the formation of dynamically recrystallized fine grains and the reprecipitation process that might take place during the cooling cycle. The TMAZ hardness gives lower values than the SZ and higher values than the HAZ. The increase in hardness of the TMAZ over the HAZ is likely due to the high dislocation density achieved by plastic deformation during the process of FSSW [14,47]. The highest hardness of the weld zones is achieved for the FSSWed joint at 600 rpm (Figure 12b). Thus, selected areas on the macrostructure of this joint (Figure 14a) were investigated using OM as a representative of the weld zone microstructure features. It can be seen that the welding zones on both sides of the keyhole center line look like a mirror and are arranged as SZ, TMAZ, and HAZ, as given in Figure 14b (left side) and Figure 14c (right side). The keyhole surrounding area is the SZ achieved during the FSSW process of the two AA6082-T6 sheets, and it is characterized by equiaxed refined grains (Figure 14e). Generally, optimizing the FSW [50,51] and the FSSW [3,36] parameters leads to grain refinement in the SZ due to dynamic recrystallization. These variations in microstructure features correspond to the variation in the hardness values in the weld zones (Figure 12b). 

Instead of incomplete W-shape hardness profiles of the FSSWed joints (Figure 12), complete W-shape hardness profiles are achieved for the RFSSWed joints (Figure 13). As mentioned before in the experimental section, the FSD process was performed at the condition of 400 rpm AA2011-T6 consumable rod rotation speed and 1 mm/min feed rate. Thus, it is expected that the deposited material (keyhole refilling material) has the same microstructure feature and mechanical properties. The average measured hardness value of the refilled keyhole is 117 ± 5 HV for all the RFSSW joints, as shown in Figure 13a–d. During the deposition process, the material of the rotating consumable rod is subjected to severe plastic deformation at high homologous temperatures. As a result, the material deposited by the friction stirring process undergoes dynamic recrystallization and develops a very fine grain size [28,29]. El-Sayed Seleman et al. [28] studied utilizing the FSD process to manufacture continuous multilayer high-performance, metal-based AA2011/nano Al_2_O_3_ composites using AA2011 in two temper conditions of AA2011-T6 and AA2011-O as a matrix. The results showed that the microstructure of the starting, consumable rods, AA2011-T6 and AA2011-O, was changed from coarse grain to refined equiaxed grain throughout the deposited material. Additionally, the size of precipitates (Al_7_Cu_2_Fe and Al_2_Cu) decreased with the stirring action during FSD for the deposited matrix and the produced composite. The dispersion and fragmentation of these intermetallics in the Al matrix have been detected in other works [29,33]. These microstructure features are gained for the deposited material in the keyholes of the FSSW joints. Figure 14f shows the microstructure of the AA6082-T6 RFSSWed joint welded at 600 rpm and 3 s with keyhole refilling by AA2011-T6 via the FSD conditions of 400 rpm and 1 mm/min. It can be seen that equiaxed refined grains (average grain size 2 ± 0.2 μm) due to the dynamic recrystallization compared with coarse grains AA2011-T6 rod BM (45 ± 8 μm) are detected. In addition, the intermetallics (Al_7_Cu_2_Fe and Al_2_Cu) are highly dispersed and fragmented. 

### 3.6. Tensile Shear Test and Fracture Surfaces

**Figure 15:**
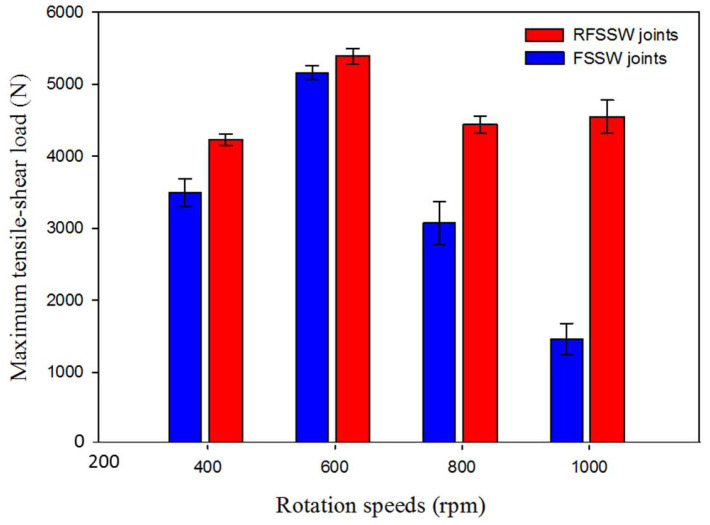
Maximum tensile-shear load of the AA6082-T6 FSSWed joints (before keyhole refilling) processed at a constant dwell time of 3 s and different rotation speeds of 400 to 1000 rpm and the RFSSW lap joints. All the keyholes of the FSSW joints refilled at the same FSD parameters of 400 rpm AA2011-T6 consumable rod rotation speed and 1 mm/min feed rate.

The tensile shear test is an important mechanical property for joint efficiency evaluation for vehicles and automotive structure designs. The tensile shear value of the spot welded joint is affected by FSSW process parameters [6,9,21,22]. The maximum tensile shear load of the FSSW and RFSSW lap joints of AA6082-T6 dissimilar sheet thickness is illustrated in Figure 15. 

**Figure 16:**
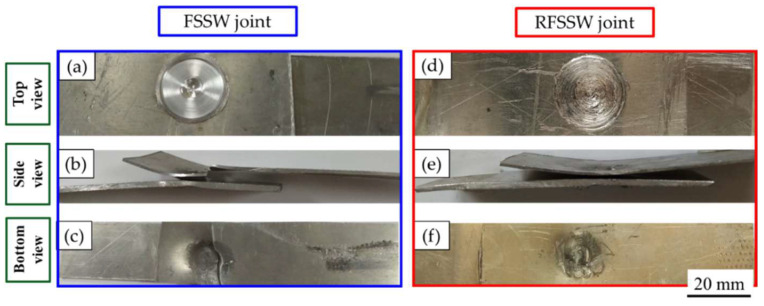
Top, side, and bottom photographs show the joint appearance of the AA6082-T6 dissimilar sheet thickness welded at 600 rpm and 3 s after tensile testing, where (**a**–**c**) the FSSW joint [36] and (**d**–**f**) the RFSSW joint are refilled with AA2011-T6 at 400 rpm and 1 mm/min.

**Figure 17:**
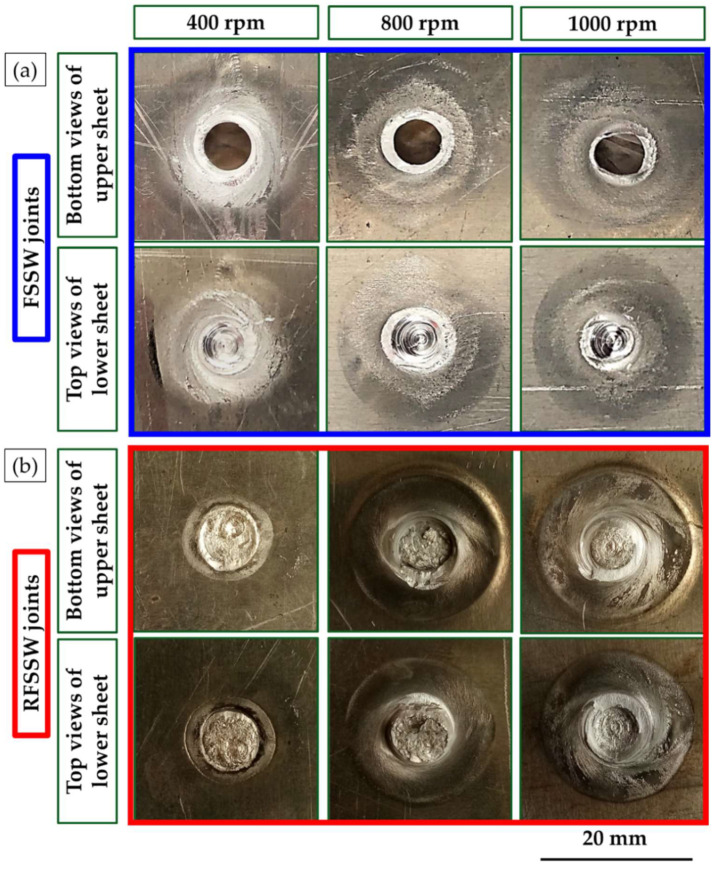
Photographs of the joints after tensile shear testing for (**a**) the FSSW lap joints welded at a dwell time of 3 s and rotation speeds of 400, 800, and 1000 rpm [36] and (**b**) the RFSSW joints welded at the same conditions and refilled at a 400 rpm AA2011-T6 consumable rod rotation speed and a 1 mm/min feed rate.

It can be noted that the FSSW joint welded at a constant dwell time of 3 s by applying a tool rotation speed of 600 rpm gives the highest tensile shear load of 4300 ± 80 N compared with that obtained by the other welded joints. The two AA6082-T6 sheets of this joint are still connected after the tensile shear testing, as given in Figure 16a–c. This indicates high joint efficiency and may be ascribed to a larger fully bonded section size and a lower hook height [36]. Besides, the enhancement in hardness in the SZ of this joint is over than that noted in the stir zones of the other spot-welded joints. In contrast, the FSSWed joints processed at the same dwell time (3 s) and different tool rotation speeds of 400, 800, and 1000 rpm are completely separated during the tensile shear testing, as given in Figure 17a. On the one hand, the failed joint FSSWed at a relatively lower rotation speed of 400 rpm may be attributed to insufficient heat input, which affects in mixing the dissimilar sheet thicknesses AA6082-T6 during the welding process. On the other hand, the tensile shear loads of the spot welds produced at higher tool rotation speeds of 800 and 1000 rpm illustrate a decrease in the maximum tensile shear load. This decrease in strength for these joints is ascribed to the increase in thermal softening in the SZ with the increasing the heat input and also to the reduced thickness of the upper sheet underneath the shoulder. Ohashi et al. [52] and Xie et al. [53] reported that the mechanical properties of the FSSW joints are mainly governed by both the weld bonded area and the upper sheet underneath the shoulder. In addition, the precipitate-free zones around the grain boundaries due to precipitate coarsening might have reduced the strength of the SZ [36]. From Figure 15, it can be also seen that all the RFSSW lap joints show higher tensile shear loads than that given by the FSSW (unrefilled FSSW) lap joints. The RFSSW joint (welded at 600 rpm/3 s and refilled at 400 rpm/1 mm/min) shows the highest tensile shear load of 5400 N ±100. Its enhancement in the tensile shear load is 25% over that recorded by the unrefilled spot joint. The two sheets of the RFSSW joint are still connected after the tensile shear testing, as given in Figure 16d–f. Furthermore, the other refilled joints show enhancements of 45%, 73%, and 273% over the unrefilled joints processed at 400, 800, and 1000 rpm, respectively. This enhancement for all the refilled joints is ascribed to the repairing of the keyhole defect by depositing high-strength AA 2011-T6 Al alloy via FSD and the strong bonding between the filling deposited material and the key hole interface, in addition to the microstructure features of the filling cavity of the keyhole. Both the deposition process and the good bonding between the keyhole interface and the FSD material filling the cavity are achieved in a solid-state process. Figure 17 displays the separated tested FSSW and RFSSW lap joint after the tensile shear test. 

**Figure 18:**
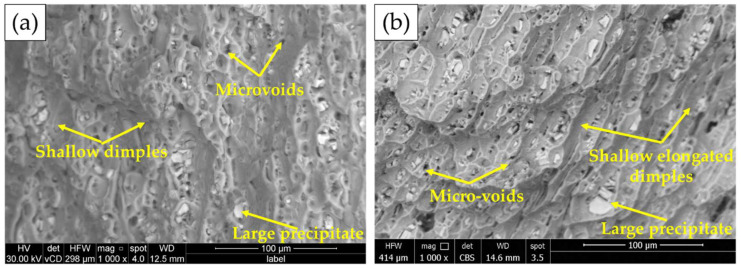
SEM images of the fracture surface of AA6082-T6 BMs: (**a**) 1 mm and (**b**) 2 mm [36].

The fracture surfaces of the BMs after tensile testing are given in Figure 18. Two fracture modes in terms of ductile and brittle fractures are detected for both the 1 and 2 mm sheet thicknesses. The Al matrix shows a ductile fracture in terms of different shallow dimple sizes. While the (Fe, Mn)_3_SiAl_l2_ and Mg_2_Si coarse precipitates show a brittle fracture. Microvoids due to the pullout of the fragmented intermetallics are also noticed on the fracture surface of the two sheets. The dimple size is related to the initial grain structure of the two sheets’ materials. 

**Figure 19:**
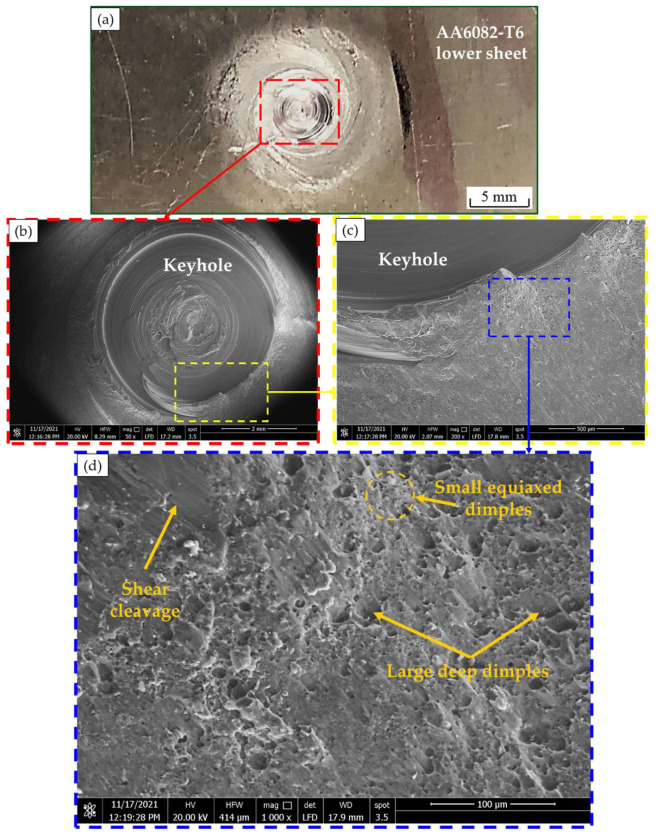
(**a**) Photograph of the top surface of the lower sheet of AA6082 -T6 FSSW (welded at 400 rpm and 3 s) after tensile shear testing and (**b**–**d**) SEM images of the fracture surface at different magnifications [36].

Figure 19a illustrates a photograph of the top surface of the lower sheet of the AA6082-T6 FSSW joint welded at a constant dwell time of 3 s and a rotation speed of 400 rpm after tensile shear testing, and Figure 19b–d shows the SEM fracture surface at different magnifications. The spot weld joint is failed by a tensile shear mechanism. Under the tensile shear loading, the crack begins at the tip of the hook of the partially bonded region and propagates preferentially in a horizontal direction at the lap joint interface, shearing the SZ, causing failure. The fracture surface of the failed region shows a typically ductile mode fracture in terms of different sizes of equiaxed deep dimples (Figure 19d) compared with shallow elongated dimples of the fracture surface of both BMs (Figure 18), indicating the grain refining of the SZ during the FSSW process. Shear cleavage is observed, indicating the direction of the tensile shear mechanism. 

**Figure 20:**
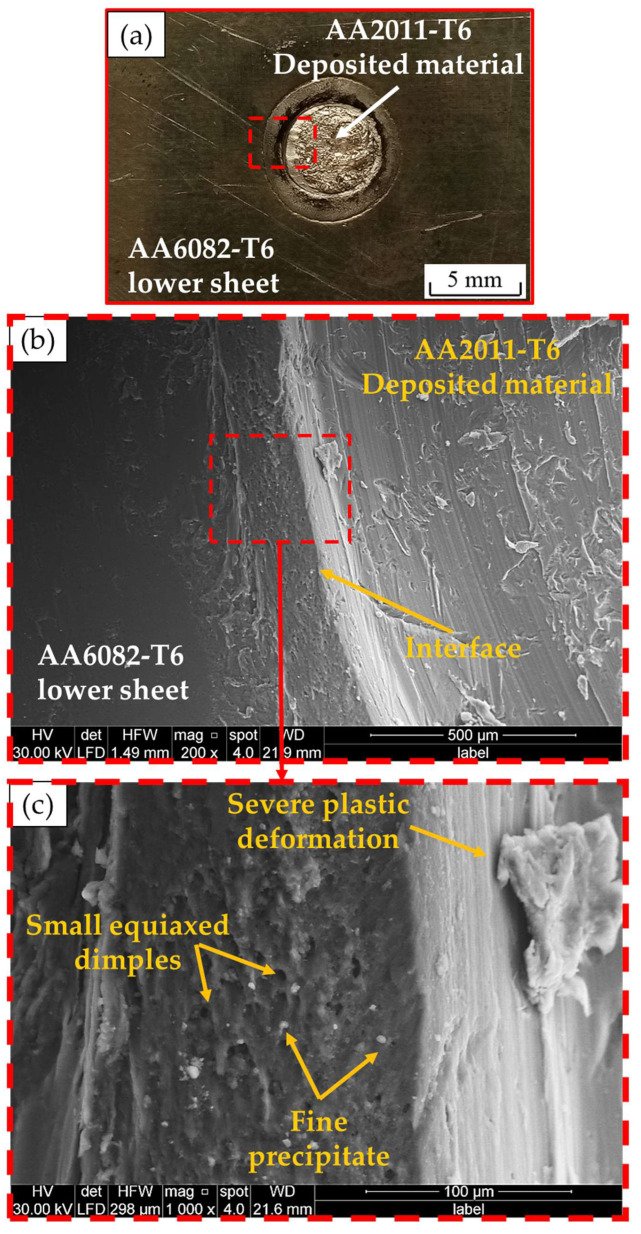
(**a**) Photograph of the top surface of the lower sheet of AA6082-T6 of the RFSSW joint (welded at 400 rpm and 3 s) after tensile shear testing and (**b**,**c**) SEM images of the fracture surface at different magnifications.

Figure 20a shows a photograph of the top surface of the lower sheet of the AA6082-T6 RFSSW joint (spot welding conditions of 400 rpm and 3 s and refilled conditions of 400 rpm and 1 mm/min) after tensile shear testing. Figure 20b,c shows the SEM fracture surface at different magnifications. The refilled joint is failed also by the tensile shear mechanism. Under the tensile shear loading, the crack begins at the tip of the hook of the partially bonded region and propagates at the lap joint interface, shearing the SZ of the welded area and the deposited refilled material, causing failure. The fracture surface of the failed material shows two distinct regions (AA6082-T6 weld sheets and AA2011-T6 deposited material) having a well-bonded interface (Figure 20b). The refilled joint reveals ductile fractures in both regions as they have equiaxed fine grain structures due to the stirring action in both the weld region and the refilled keyhole (Figure 20b).

## 4. Conclusions

In this study, an innovative technique was developed based on the friction stir deposition and examined to refill the keyhole of the FSSWed aluminum samples. A comparative investigation and evaluation were carried out between the unrefilled FSSWed and RFSSWed samples. Based on that, the following conclusions can be outlined:

1. The applied FSSW parameters in terms of different rotational speeds of 400 to 1000 rpm and a constant dwell time of 3 s succeeded in spot-welding two different thin sheet thicknesses of AA6082-T6.
2. The applied FSD parameters in terms of a feed rate of 1 mm/min and an AA2011-T6 consumable rod rotational speed of 400 rpm succeeded in refilling FSSW keyholes and shoulder projections of all the produced AA6082-T6 FSSW lap joints with defect-free continuous multilayers.
3. All the RFSSW joints show higher bearing tensile shear loads than that given by the as-FSSWed joints.
4. Among all the RFSSW joints, the RFSSW joint (welded at 600 rpm/3 s and refilled at 400 rpm/1 mm/min) promotes the highest tensile shear load of 5400 N ± 100. Meanwhile, among all the FSSW joints, the FSSW joint (welded at 600 rpm/3 s) gives the highest tensile shear load of 4300 N ± 80.
5. The suggested FSD technique, including the consumable tool design and the FSD parameters, open new horizons for repairing the FSSW keyhole defect for different welded joints.

## Figures and Tables

**Table 1:** The chemical composition (in wt.%) of AA6082-T6 sheets and AA2011-T6 rods.

Element	Si	Mg	Fe	Mn	Zn	Cr	Ti	Cu	Bi	Pb	Al
AA6082-T6	0.75	0.60	0.50	0.40	0.20	0.20	0.10	0.10	-	-	Bal
AA2011-T6	0.09	-	0.37	-	0.06	0.04	0.03	4.83	0.25	0.3	Bal

**Table 2:** The peak measured temperature during the FSSW process.

Rotational speeds (rpm)	400	600	800	1000
Temperature (°C)	225 ± 2	245 ± 3	300 ± 5	350 ± 2

## Data Availability

Data will be available upon request through the corresponding author.

## References

[1] Suryanarayanan R., Sridhar V.G. (2020). Effect of Process Parameters in Pinless Friction Stir Spot Welding of Al 5754-Al 6061 Alloys. Metallogr. Microstruct. Anal..

[2] Muhayat N., Priatmana Putra B., Triyono (2019). Mechanical Properties and Microstructure of Friction Stir Spot Welded 6082-T6 Aluminium Alloy Joint. MATEC Web Conf..

[3] Ahmed M.M.Z., Ahmed E., Hamada A.S., Khodir S.A., El-Sayed Seleman M.M., Wynne B.P. (2016). Microstructure and Mechanical Properties Evolution of Friction Stir Spot Welded High-Mn Twinning-Induced Plasticity Steel. Mater. Des..

[4] Ahmed M.M.Z., Habba M.I.A., Jouini N., Alzahrani B., El-Sayed Seleman M.M., El-Nikhaily A. (2021). Bobbin Tool Friction Stir Welding of Aluminum Using Different Tool Pin Geometries: Mathematical Models for the Heat Generation. Metals.

[5] Ahmed M.M.Z., Ataya S., El-Sayed Seleman M.M., Ammar H.R., Ahmed E. (2017). Friction Stir Welding of Similar and Dissimilar AA7075 and AA5083. J. Mater. Process. Technol..

[6] Ahmed M.M.Z., Abdul-Maksoud M.A.A., El-Sayed Seleman M.M., Mohamed A.M.A. (2022). Effect of Dwelling Time and Plunge Depth on the Joint Properties of the Dissimilar Friction Stir Spot Welded Aluminum and Steel. J. Eng. Res..

[7] Ahmed M.M.Z., Ataya S., El-Sayed Seleman M.M., Mahdy A.M.A., Alsaleh N.A., Ahmed E. (2021). Heat Input and Mechanical Properties Investigation of Friction Stir Welded Aa5083/Aa5754 and Aa5083/Aa7020. Metals.

[8] Ahmed M.M.Z., El-Sayed Seleman M.M., Zidan Z.A., Ramadan R.M., Ataya S., Alsaleh N.A. (2021). Microstructure and Mechanical Properties of Dissimilar Friction Stir Welded AA2024-T4/AA7075-T6 T-Butt Joints. Metals.

[9] Ataya S., Ahmed M.M.Z., El-Sayed Seleman M.M., Hajlaoui K., Latief F.H., Soliman A.M., Elshaghoul Y.G.Y., Habba M.I.A. (2022). Effective Range of FSSW Parameters for High Load-Carrying Capacity of Dissimilar Steel A283M-C/Brass CuZn40 Joints. Materials.

[10] Deng L., Li S., Ke L., Liu J., Kang J. (2019). Microstructure and Fracture Behavior of Refill Friction Stir Spot Welded Joints of AA2024 Using a Novel Refill Technique. Metals.

[11] Janga V.S.R., Awang M., Yamin M.F., Suhuddin U.F.H., Klusemann B., Dos Santos J.F. (2021). Experimental and Numerical Analysis of Refill Friction Stir Spot Welding of Thin AA7075-T6 Sheets. Materials.

[12] Shi Y., Yue Y., Zhang L., Ji S., Wang Y. (2018). Refill Friction Stir Spot Welding of 2198-T8 Aluminum Alloy. Trans. Indian Inst. Met..

[13] Fratini L., Barcellona A., Buffa G., Palmen D. (2007). Friction Stir Spot Welding of AA6082-T6: Influence of the Most Relevant Process Parameters and Comparison with Classic Mechanical Fastening Techniques. Proc. Inst. Mech. Eng. Part B J. Eng. Manuf..

[14] Aydin H., Tuncel O., Umur Y., Tutar M., Bayram A. (2017). Effect of Welding Parameters on Microstructure and Mechanical Properties of Aluminum Alloy AA6082-T6 Friction Stir Spot Welds. Indian J. Eng. Mater. Sci..

[15] Tozaki Y., Uematsu Y., Tokaji K. (2010). A Newly Developed Tool without Probe for Friction Stir Spot Welding and Its Performance. J. Mater. Process. Technol..

[16] Khosa S.U., Weinberger T., Enzinger N. (2010). Thermo-Mechanical Investigations during Friction Stir Spot Welding (FSSW) of AA6082-T6. Weld. World.

[17] Habeeb S.S., Katratwar T. (2016). Analyzing Process of Friction Stir Spot Weld Joint. Int. J. Sci. Technol. Eng..

[18] Pan T.Y. (2007). Friction Stir Spot Welding (FSSW)—A Literature Review. SAE Tech. Pap..

[19] Haneklaus N., Cionea C., Reuven R., Frazer D., Hosemann P., Peterson P.F. (2016). Hybrid Friction Diffusion Bonding of 316L Stainless Steel Tube-to-Tube Sheet Joints for Coil-Wound Heat Exchangers. J. Mech. Sci. Technol..

[20] Suryanarayanan R., Sridhar V.G. (2020). Studies on the Influence of Process Parameters in Friction Stir Spot Welded Joints—A Review. Mater. Today Proc..

[21] Silva B.H., Zepon G., Bolfarini C., dos Santos J.F. (2020). Refill Friction Stir Spot Welding of AA6082-T6 Alloy: Hook Defect Formation and Its Influence on the Mechanical Properties and Fracture Behavior. Mater. Sci. Eng. A.

[22] Xu Z., Li Z., Ji S., Zhang L. (2018). Refill Friction Stir Spot Welding of 5083-O Aluminum Alloy. J. Mater. Sci Technol..

[23] Singh B., Upadhyaya R. (2021). Influence of Flat Friction Stir Spot Welding Process Parameters on Quality Characteristics of AA 6082 Weld. J. Univ. Shanghai Sci. Technol..

[24] Jambhale S., Kumar S., Kumar S. (2022). Characterization and Optimization of Flat Friction Stir Spot Welding of Triple Sheet Dissimilar Aluminium Alloy Joints. Silicon.

[25] Feng X.S., Li S.B., Tang L.N., Wang H.M. (2020). Refill Friction Stir Spot Welding of Similar and Dissimilar Alloys: A Review. Acta Metall. Sin..

[26] Tier M.D., Rosendo T.S., Dos Santos J.F., Huber N., Mazzaferro J.A., Mazzaferro C.P., Strohaecker T.R. (2013). The Influence of Refill FSSW Parameters on the Microstructure and Shear Strength of 5042 Aluminium Welds. J. Mater. Process. Technol..

[27] Zhao Y., Wang C., Li J., Tan J., Dong C. (2018). Local Melting Mechanism and Its Effects on Mechanical Properties of Friction Spot Welded Joint for Al-Zn-Mg-Cu Alloy. J. Mater. Sci. Technol..

[28] El-Sayed Seleman M.M., Ataya S., Ahmed M.M.Z., Hassan A.M.M., Latief F.H., Hajlaoui K., El-Nikhaily A.E., Habba M.I.A. (2022). The Additive Manufacturing of Aluminum Matrix Nano Al_2_O_3_ Composites Produced via Friction Stir Deposition Using Different Initial Material Conditions. Materials.

[29] Ahmed M.M.Z., El-Sayed Seleman M.M., Elfishawy E., Alzahrani B., Touileb K., Habba M.I.A. (2021). The Effect of Temper Condition and Feeding Speed on the Additive Manufacturing of AA2011 Parts Using Friction Stir Deposition. Materials.

[30] Alzahrani B., Seleman M.M.E.S., Ahmed M.M.Z., Elfishawy E., Ahmed A.M.Z., Touileb K., Jouini N., Habba M.I.A. (2021). The Applicability of Die Cast A356 Alloy to Additive Friction Stir Deposition at Various Feeding Speeds. Materials.

[31] Elfishawy E., Ahmed M.M.Z., El-Sayed Seleman M.M. (2020). Additive Manufacturing of Aluminum Using Friction Stir Deposition. Minerals, Metals and Materials Series.

[32] Perry M.E.J., Griffiths R.J., Garcia D., Sietins J.M., Zhu Y., Yu H.Z. (2020). Morphological and Microstructural Investigation of the Non-Planar Interface Formed in Solid-State Metal Additive Manufacturing by Additive Friction Stir Deposition. Addit. Manuf..

[33] Dilip J.J.S., Janaki Ram G.D. (2013). Microstructure Evolution in Aluminum Alloy AA 2014 during Multi-Layer Friction Deposition. Mater. Charact..

[34] Priedeman J.L., Phillips B.J., Lopez J.J., Tucker Roper B.E., Hornbuckle B.C., Darling K.A., Jordon J.B., Allison P.G., Thompson G.B. (2020). Microstructure Development in Additive Friction Stir-Deposited Cu. Metals.

[35] Ahmed M.M.Z., El-Sayed Seleman M.M., Shazly M., Attallah M.M., Ahmed E. (2019). Microstructural Development and Mechanical Properties of Friction Stir Welded Ferritic Stainless Steel AISI 409. J. Mater. Eng. Perform..

[36] Ahmed M.M.Z., Seleman M.M.E.S., Ahmed E., Reyad H.A., Touileb K., Albaijan I. (2022). Friction Stir Spot Welding of Different Thickness Sheets of Aluminum Alloy AA6082-T6. Materials.

[37] Hirasawa S., Badarinarayan H., Okamotoc K., Tomimura T., Kawanami T. (2010). Analysis of Effect of Tool Geometry on Plastic Flow During Friction Stir Spot Welding Using Particle Method. J. Mater. Process. Technol..

[38] Atak A., Şık A., Özdemir V. (2018). Thermo-Mechanical Modeling of Friction Stir Spot Welding and Numerical Solution with the Finite Element Method. Int. J. Eng. Appl. Sci..

[39] Kumar R.R., Kumar A., Kumar S. (2018). Effect on Tool Design and Heat Input of Some Welding Parameters in Friction Stir Welded Interstitial Free Steels. Int. J. Eng. Technol. Innov..

[40] John J., Shanmughanatan S.P., Kiran M.B. (2018). ScienceDirect Effect of Tool Geometry on Microstructure and Mechanical Properties of Friction Stir Processed AA2024-T351 Aluminium Alloy. Mater. Today Proc..

[41] John J., Shanmuganatan S.P., Kiran M.B., Kumar V.S.S. (2018). Investigation of Friction Stir Processing Effect on AA 2014-T6. Mater. Manuf. Process..

[42] Ma Z.Y. (2008). Friction Stir Processing Technology: A Review. Metall. Mater. Trans. A.

[43] Chaudhary B., Jain N.K., Murugesan J. (2022). Development of Friction Stir Powder Deposition Process for Repairing of Aerospace-Grade Aluminum Alloys. CIRP J. Manuf. Sci. Technol..

[44] Zhang Z., Tan Z.J., Li J.Y., Zu Y.F., Liu W.W., Sha J.J. (2019). Experimental and Numerical Studies of Re-Stirring and Re-Heating Effects on Mechanical Properties in Friction Stir Additive Manufacturing. Int. J. Adv. Manuf. Technol..

[45] Moran J., Sucharitakul T. (2015). Variations in Dry Sliding Friction Coefficients with Velocity. Recent Adv. Mech. Mater. Mech. Eng. Chem. Eng..

[46] Gandra J., Krohn H., Miranda R.M., Vilaça P., Quintino L., Dos Santos J.F. (2014). Friction Surfacing—A Review. J. Mater. Process. Technol..

[47] Aydın H., Tunçel O., Tutar M., Bayram A. (2021). Effect of Tool Pin Profile on the Hook Geometry and Mechanical Properties of a Friction Stir Spot Welded Aa6082-T6 Aluminum Alloy. Trans. Can. Soc. Mech. Eng..

[48] Ma C., Hou L., Zhang J., Zhuang L. (2016). Influence of Thickness Reduction per Pass on Strain, Microstructures and Mechanical Properties of 7050 Al Alloy Sheet Processed by Asymmetric Rolling. Mater. Sci. Eng. A.

[49] Ahmed M.M.Z., Wynne B.P., Rainforth W.M., Addison A., Martin J.P., Threadgill P.L. (2019). Effect of Tool Geometry and Heat Input on the Hardness, Grain Structure, and Crystallographic Texture of Thick- Section Friction Stir-Welded Aluminium. Metall. Mater. Trans. A.

[50] Ahmed M.M.Z., El-Sayed Seleman M.M., Touileb K., Albaijan I., Habba M.I.A. (2022). Microstructure, Crystallographic Texture, and Mechanical Properties of Friction Stir Welded Mild Steel for Shipbuilding Applications. Materials.

[51] Seleman M.M.E.S., Ahmed M.M.Z., Ramadan R.M., Zaki B.A. (2022). Effect of FSW Parameters on the Microstructure and Mechanical Properties of T-Joints between Dissimilar Al-Alloys. Int. J. Integr. Eng..

[52] Ohashi R., Fujimoto M., Mironov S., Sato Y.S., Kokawa H. (2009). Effect of Contamination on Microstructure in Friction Stir Spot Welded DP590 Steel. Sci. Technol. Weld. Join..

[53] Xie G.M., Cui H.B., Luo Z.A., Yu W., Ma J., Wang G.D. (2016). Effect of Rotation Rate on Microstructure and Mechanical Properties of Friction Stir Spot Welded DP780 Steel. J. Mater. Sci. Technol..

